# A case of dual left anterior descending artery with myocardial infarction

**DOI:** 10.4322/acr.2020.223

**Published:** 2020-11-20

**Authors:** Abhaykumar Binodkumar Dheeraj, Sandeep Kumar Giri, Pankaj Suresh Ghormade

**Affiliations:** 1 All India Institute of Medical Sciences, Department of Forensic Medicine and Toxicology, Raipur, Chhattisgarh, India

**Keywords:** Anomalous Left Coronary artery, Myocardial Infarction, Autopsy

## Abstract

The Left anterior descending artery (LAD) is a branch of the left main coronary artery which runs obliquely towards the apex of the heart in the anterior interventricular sulcus. Among all of the coronary arteries, the LAD artery has the most constant course. Amongst the anomalies of coronaries, the duplication or bifurcation of the LAD artery is infrequent. The classification of the bifurcation of the LAD has been extensively described in various reports with the widespread use of CT Angiography. We describe herein, an anomalous LAD detected on autopsy. This unusual case highlights the gross autopsy finding of Type-I anomalous dual LAD coronary artery in a young adult who died of Myocardial Infarction.

## INTRODUCTION

The Left anterior descending (LAD) artery is a branch of the left main coronary artery, which runs obliquely towards the apex of the heart in the anterior interventricular sulcus.^1^ The dual LAD coronary artery is defined as two separate vessels in the anterior interventricular sulcus (AIS) of the heart.^2^ In 1983, Spindola-Franco et al.^3^ first described and classified the LAD artery anomalies into 4 types by using Coronary Catheter Angiography. The literature search revealed additional new types based upon the origin, course, and location of the long LAD artery, further extending its classification.^2^ ^,^ ^4^ ^-^ ^7^ In most cases, the presence of dual LAD was detected on CT-angiography in patients presented with complaints of angina and chest pain.^2^ ^,^ ^4^ ^-^ ^7^

Nikolić et al.^8^ stated, in their autopsy study, that myocardial bridging of coronary arteries in type-3 dual LAD has a protective role in the development of atherosclerosis, though the probability of developing atherosclerosis is equal in both the arteries of dual LAD. However, this case describes a Type-1 dual LAD and it highlights the role of the location of critical coronary atherosclerotic stenosis, or the occlusive thrombus, in the development of myocardial infarction despite the presence of dual LAD.

## CASE REPORT

A 30-year-old male patient was brought to our Tertiary Care Institute with the chief complaint of intermittent epigastric pain over the last day. On clinical examination, he was unconscious; blood pressure was not recordable, pulses were non-palpable, oxygen saturation was zero, heart sounds not audible on auscultation, and electrocardiogram was flat-lined. As a result, he was declared dead. A medicolegal autopsy was ordered to know the cause of death.

As per history provided by the parents, the deceased had no significant history or any symptom suggestive of an underlying cardiac disease, diabetes mellitus, hypertension, chronic kidney disease, or any other endocrinal disorders. He was a smoker, tobacco chewer, and alcoholic over the last 7 years. He was not on any regular medications.

## AUTOPSY FINDINGS

The Body mass index was 21, and the post-mortem interval was ten hours. External examination revealed no injuries over the body.

**Figure 1:**
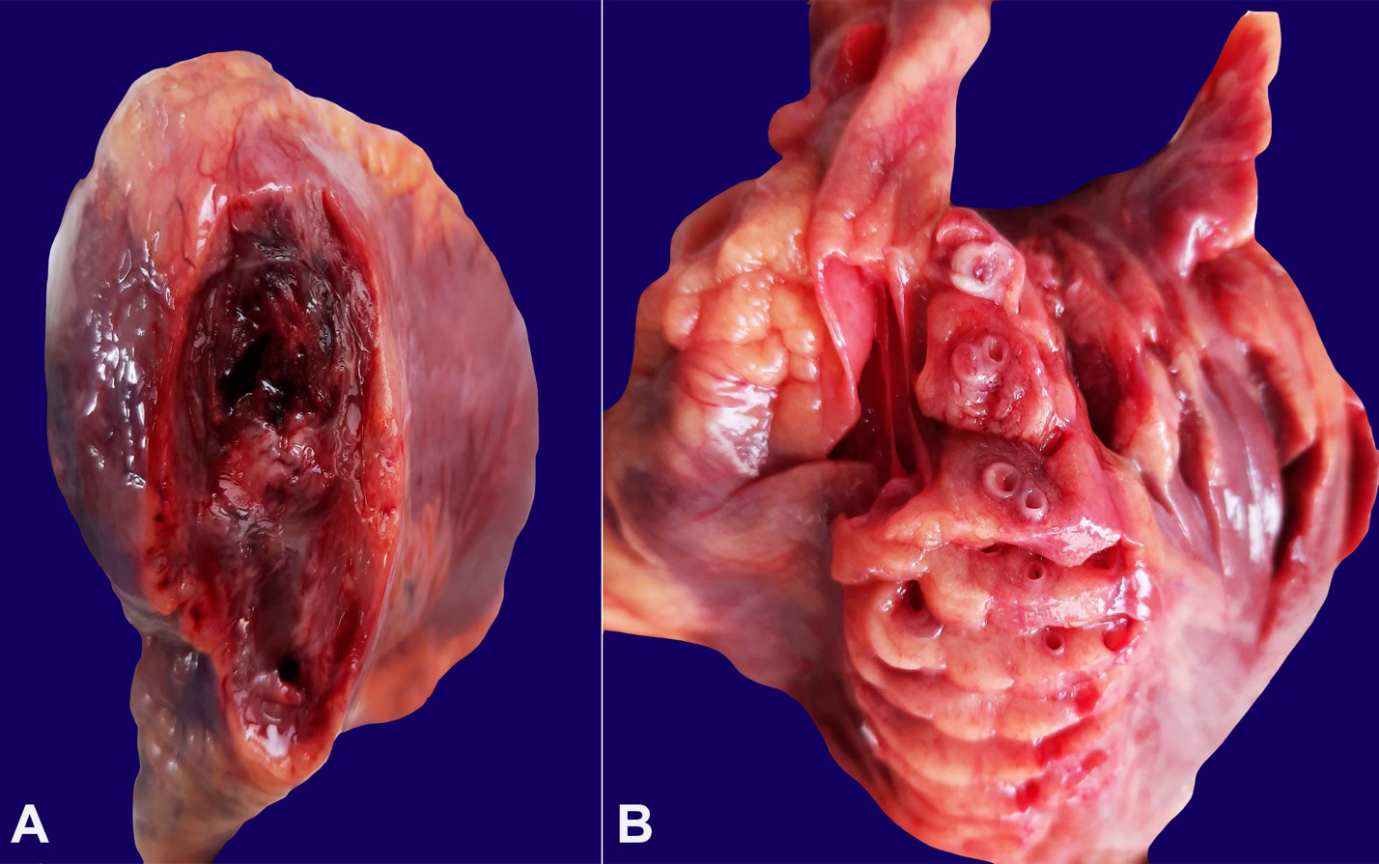
Gross view of the cut section of the heart depicting in **A –** dark mottled area consistent with a trans-mural MI taken; **B –** LAD proper and at its bifurcation show a fibrous cap fibroatheroma (AHA Grade 6) with luminal narrowing of about 80 to 90% with overlying plaque erosion.

On internal examination, finding in all organs were unremarkable except the heart, which weight was 296 grams (reference range [RR]; mean 302 g). The gross heart inspection showed a reddish-brown patch consistent with a myocardial infarct (MI), measuring 2 cm × 1.5 cm over the apex of the left ventricle. On dissection, the myocardium at the injured level showed a transmural dark mottled area (Figure 1A). On sectioning of coronaries, the proximal segment of the left anterior descending (LAD) proper has a bifurcation leading to two anterior descending arteries, right and left in its further course within the interventricular septum (Figure 1B).

Atherosclerotic luminal narrowing of about 80 to 90% with a fibrous cap fibroatheroma (AHA Grade 6) with overlying plaque erosion in the LAD proper just before its bifurcation in the two anterior descending arteries. The right-sided LAD (short LAD) terminated proximal to inter-ventricular septum, while the left one followed the course along the inter-ventricular septum. The right-sided LAD showed a luminal narrowing of about 60%, whereas long LAD and remaining coronaries were patent without any anomalies. Both lungs were edematous, weighing 498 g (mean RR; 450 g) the right lung, and 551 g (mean RR; 375 g) the left one.

**Figure 2:**
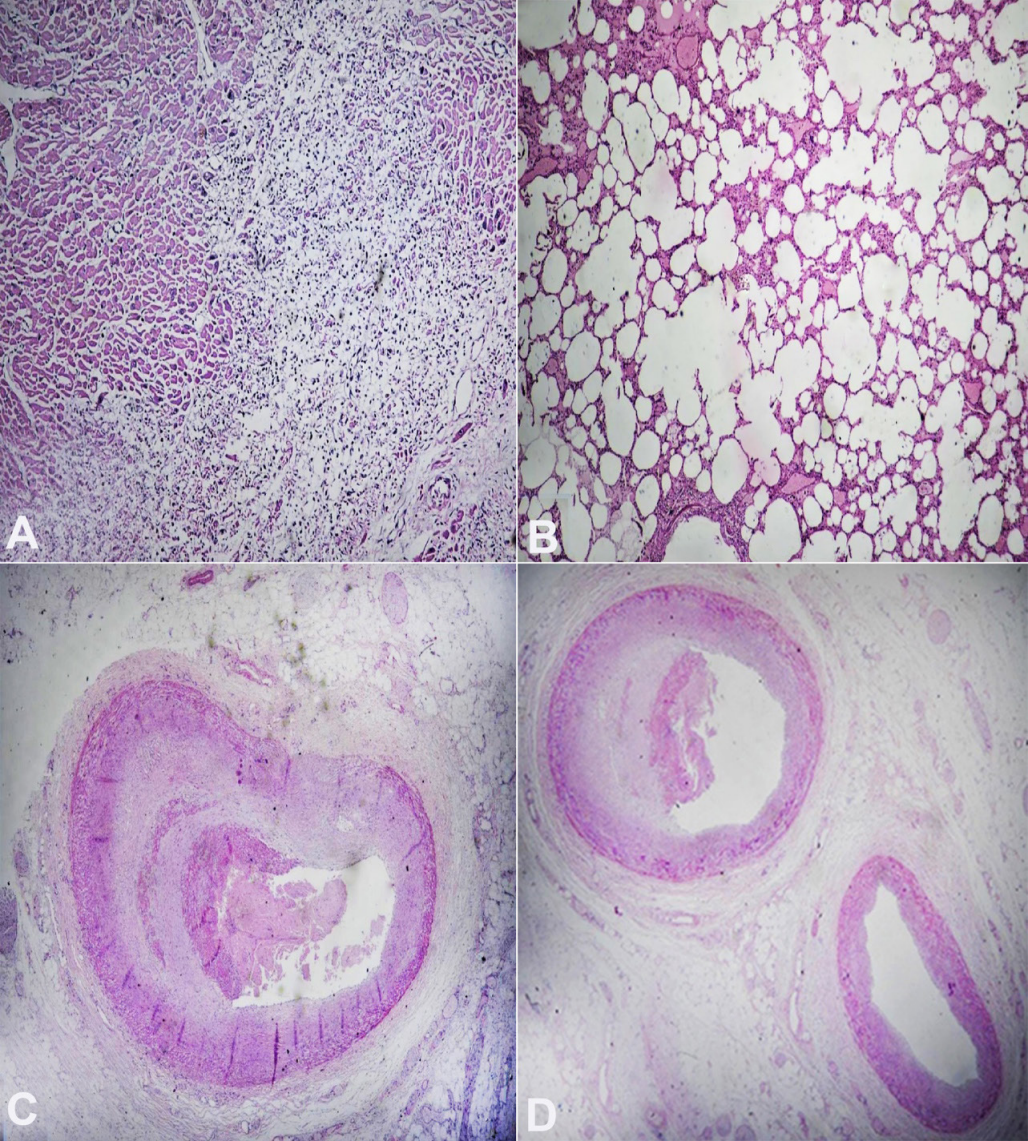
Photomicrographs of the myocardium in A, lung in B, coronaries in C and D. **A –** shows recent infarction with early granulation tissue formation at the edge of the infarct; **B –** lung with diffuse alveolar damage and emphysematous changes; **C –** shows atherosclerosis with a near occlusive recent thrombus of LAD proper; and **D –** shows atherosclerosis with mural thrombus in the short LAD.

Histopathological examination of the heart depicted recent infarction with early granulation tissue formation at the edge of the infarct (Figure 2A); atherosclerosis with a near occlusive recent thrombus of LAD proper (Figure 2C); atherosclerosis with mural thrombus in the short LAD (Figure 2D). Hence, the cause of the death of our case was opined as MI due to coronary artery disease with dual LAD. Figure 2B depicts edema, diffuse alveolar damage with emphysematous changes.

## DISCUSSION

The LAD coronary artery is an arterial branch arising from the main left coronary artery, which originates from the left aortic sinus of the ascending aorta. Usually, LAD passes between the pulmonary trunk and the left atrial appendage and descends obliquely towards the apex of the heart. It may give rise to some diagonal branches across the anterior surface of left ventricle during its course.^1^

In 1983, Spindola-Franco et al.^3^ first described and classified anomalies of LAD artery by the use of Coronary Catheter Angiography. First four types were classified on the basis of origin and course of long LAD. In first three types, LAD proper consists of a short branch that ends high in the anterior interventricular groove and a long branch that most commonly originates as its early branch. In type-1, long and short LAD originate from the LAD proper. The short LAD run in the AIVS and terminate abruptly long before reaching the apex. The long LAD after leaving the AIVS ran on the epicardial anterior surface of the left ventricle. Its distal third re-entered the sulcus and continued to the apex. In type-2, the long LAD runs over the anterior wall of the right ventricle, rest findings were similar to type-1. In type-3, the long LAD runs intramyocardially, rest similar to type-1. In type-4 cases long LAD originates from RCA^3^ (Table 1).

**Table 1:** Classification of the dual left anterior descending artery

Type of dual LAD	LAD Proper	Short LEFT ANTERIOR DESCENDING ARTERY		Long LAD	
		Origin	Termination	Origin	Course and Location
Type-1^3^	Present	From LAD proper	In proximal AIVS	From LAD proper	In proximal AIVG epicardially on LV side, re-enters the distal AIVS
Type-2^3^	Present	From LAD proper	In proximal AIVS	From LAD proper	In proximal AIVG epicardially on RV side, re-enters the distal AIVS
Type-3^3^	Present	From LAD proper	In proximal AIVS	From LAD proper	In proximal AIVG intramyocardially, re-enters epicardially in the distal AIVS
Type-4^3^	Absent	From LMCA	In proximal AIVS	From RCA	In proximal AIVS epicardially along anterior to RVOT, re-enters epicardially in the distal AIVS
Type-5^5^	Absent	From LCS	In proximal AIVS	From RCS	Intramyocardially within septal crest, re-enters epicardially in the distal AIVS
Type-6^6^	Absent	From LMCA	In proximal AIVS	From RCA	Epicardially between RVOT and aortic root, continuing to distal AIVS
Type-7^7^	Absent	From LMCA	In proximal AIVS	From RCS	Intramyocardially in septal crest, emerging epicardially in distal AIVS
Type-8^2^	Absent	From LMCA	In proximal AIVS	From mid RCA	Epicardially in inferior surface of RV, re-enters epicardially in the distal AIVS
Type-9^2^	Present	From LAD proper	In mid AIVS	From LAD proper	Epicardially on LV side, re-enters epicardially in the distal &terminates before reaching the apex.

AIVS: anterior interventricular sulcus; AIVG: anterior interventricular groove; LAD: left anterior descending artery; LMCA: left main coronary artery; LV: left ventricle; RCA: right coronary artery; RV: right ventricle; RVOT: right ventricular outflow tract; RCS: right coronary sinus.

An autopsy based prospective study was performed on approximately 3000-3500 hearts by Nikolić et al.^8^ They found 10 cases of type 3 dual LAD. They concluded that the atherosclerotic changes were more prominent and severe in the short rather than in the long dual LAD. Similar finding in coronaries was observed in the present case. They also established that myocardial bridging had protective role in Type-3 dual LAD in the development of atherosclerosis in both short and long LAD with due consideration to the equally acting modifiable and non-modifiable factors.

In 2008, Agarwal and Kazerooni^4^ reported two cases; the first patient was a 34-year old woman with supraventricular tachycardia and second was a 54-year-old man with atypical chest pain. Neither of the patients had notable atherosclerotic coronary artery disease in the dual left anterior descending system using the ECG-gated 64-MDCT coronary angiography. One case had type-1, and the other case had a Type-4 dual LAD artery. Whereas, in type-4 dual LAD, the long branch originates from the right coronary artery, followed an anomalous course, and enters the anterior interventricular groove.

Manchanda et al.,^5^ reported another variant, in a 29-year-old male with atypical chest pain. Physical examination, electrocardiogram, and cardiac enzymes were normal. In CT Angiography, the short LAD artery was originated independently from the left coronary sinus, and the long LAD artery originated from the right coronary sinus, having an intramyocardial route before reaching the distal interventricular groove. This finding was considered as a new variant and classified as Type-V.

Maroney and Klein^6^ reported a case of 48-year-old male with pervious history of coronary artery disease, previous angioplasty, hyperlipidemia, and generalized anxiety disorder with atypical chest pain. They found a novel type of dual LAD artery in which its long branch originated from the right coronary artery and followed a unique route underneath the right ventricular outflow tract in the interventricular septal area to the anterior interventricular groove. They proposed this new variant of the dual LAD artery as Type-VI.

In 2015, Saglam et al.^7^ examined a 53 years old male that presented a history of chest pain and found a novel dual LAD artery anomaly, which was classified as Type-VII. In this type, a long LAD artery originated from the right coronary sinus and coursed intramyocardially within the septal crest, and entered the anterior interventricular groove. In this variant, the short LAD artery originated from the left main coronary artery and terminated high in the anterior interventricular groove.

Two new variants were found by Bozlar et al.,^2^ which were classified as Type-VIII and Type-IX of the dual left anterior descending artery. In Type-VIII, the short LAD originated from LMCA and terminated in the proximal anterior interventricular groove. Long LAD originated from the mid-RCA and coursed epicardially in the inferior surface of the right ventricle (RV) and re-entered epicardially in the distal anterior interventricular groove. In Type- IX, both branches originated from the LAD proper, the short branch ended in the mid anterior interventricular groove, and the long one coursed epicardially on the left ventricle, re-entered in the distal part and ended before reaching the apex.

The aforementioned classification of the anomalous dual LAD is based upon the CT angiography findings. A literature search over a period of last 30 years was done using the keywords anomalous left coronary artery, myocardial infarction, autopsy, dual left anterior descending artery, CT angiography, pathological findings, using PubMed, MeSH, Scopus, DOAJ, Google Scholar and Copernicus provided neither a study nor a report on the anomalous dual LAD with myocardial infarction detected on autopsy.

In our case, the left aortic ostia gave rise to the left main coronary artery, which then divided to give rise to left circumflex and LAD proper. The proximal part of LAD proper then bifurcated into two anterior descending arteries, right and left in its further course. LAD’s right branch terminated proximal to interventricular septum, i.e., short LAD, while the left one coursed in the epicardium along the interventricular septum on the left ventricle side, i.e., long LAD. As per the above-cited and discussed classification, the current discussed case falls under Type-1 classification.

It has been noted in the present case that, if the atherosclerosis or thrombus originates in the proximal part or above the bifurcation of LAD proper, then the dual LAD is not protective against the development of AMI.

## CONCLUSION

Aided by new imaging techniques and therefore medical acknowledgement, new variants of the dual LAD could be described are learned. In this case, a young adult died due to MI without clinical suspicion of this entity, much probably because of his epidemiological data. This case taught us a rare presentation of AMI with dual LAD and non critical atherosclerotic lesions.
